# Hypervirulence Characteristics of Spaceflight-Mutated *Beauveria bassiana* Isolate for Integrated Control of Sweet Potato Foliar and Soil Pests

**DOI:** 10.3390/insects17070720

**Published:** 2026-07-12

**Authors:** Yijia Liu, Yuan Liu, Hongyu Gong, Zhaoxia Feng, Rongchan Li, Wei Di, Junhong Qiu, Baoli Qiu, Da Ou

**Affiliations:** 1 Engineering Research Center of Biotechnology for Active Substances, Ministry of Education, College of Life Sciences, Chongqing Normal University, Chongqing 401331, China; 2 Chongqing Foreign Language School, Chongqing 400039, China

**Keywords:** *Beauveria bassiana*, *Cylas formicarius*, *Bemisia tabaci*, spaceflight mutagenesis, ecological adaptations, integrated pest management

## Abstract

The ecological adaptations of insect pests, such as multi-niche colonization and physiological resistance to conventional chemicals, pose severe challenges to the sustainable production of sweet potato (*Ipomoea batatas*). The tobacco whitefly, *Bemisia tabaci* (Hemiptera: Aleyrodidae), and the sweet potato weevil, *Cylas formicarius* (Coleoptera: Brentidae), form a highly destructive, spatially separated pest complex. In this study, we evaluated the dual-niche pathogenicity of a *Beauveria bassiana* (Hypocreales: Cordycipitaceae) isolate, BbCF-2, generated via spaceflight mutagenesis, against *C. formicarius* and *B. tabaci* under controlled laboratory conditions. The mutated strain exhibited enhanced colony expansion and a high sporulation capacity (2.72 × 10^8^ conidia/mL). Bioassays revealed that BbCF-2 possesses significantly increased virulence compared to the wild-type strain, capable of overcoming the distinct physiological and physical barriers of both targeted pests. Against the highly sclerotized subterranean *C. formicarius* adults, BbCF-2 achieved 92.68% mortality at 15 days post-inoculation at 1 × 10^8^ conidia/mL, with an LC50 of 8.452 × 10^3^ conidia/mL and an LT50 of 6.305 days. Concurrently, against the canopy-dwelling *B. tabaci*, the isolate demonstrated rapid lethal mycosis with an LT50 of 6.718 days, effectively reducing the adult vector population prior to their typical dispersal timeframe. These results demonstrate that the spaceflight-mutated BbCF-2 strain exhibits broad pathogenicity. By simultaneously targeting both foliar and soil-dwelling pests, this single-agent biological control strategy shows potential for integrated pest management, pending greenhouse and field evaluation.

## Simple Summary

Sweet potatoes are threatened by two major pests that have adapted to modern agriculture by occupying distinct physical areas: the virus-transmitting whitefly above ground and the root-boring weevil below ground. This physical separation is a powerful survival strategy that conventional chemical sprays often fail to overcome. This study tested a novel fungal strain, a specific type of *Beauveria bassiana*, developed through spaceflight mutation, to determine if its enhanced traits could break through these defensive strategies. In laboratory experiments, we exposed both pests to the mutated fungus. The fungus was highly effective, producing high yields of conidia and rapidly killing both the sap-sucking whiteflies and the hard-shelled weevils. Our findings demonstrate that this single biological agent can simultaneously counteract the natural defenses and survival behaviors of these diverse pests, offering an innovative and sustainable solution for crop protection and global food security.

## 1. Introduction

Sweet potatoes (*Ipomoea batatas* (L.) Lam) play a crucial role in ensuring global food security, providing vital industrial raw materials, and serving as a sustainable bioethanol feedstock [1,2]. Despite advances in breeding and genomics to improve agronomic traits [3,4], their sustainable production globally is severely constrained by a highly destructive, multi-layered pest ecosystem [5,6]. Unlike crops plagued primarily by single-niche canopy pests, sweet potato fields face a dual-niche ecological threat: the tobacco whitefly, *Bemisia tabaci* (Gennadius) (Hemiptera: Aleyrodidae), aboveground, and the sweet potato weevil, *Cylas formicarius* Fabricius (Coleoptera: Brentidae), belowground [7,8]. *B*. *tabaci* causes direct phloem-feeding damage and acts as a hyper-efficient vector for devastating plant viruses, such as sweet potato virus disease (SPVD), leading to rapid epidemiological outbreaks [9,10]. Concurrently, *C*. *formicarius* larvae feed cryptically within the storage roots, forming tunnels that lead the tuber to emit a foul odor, rot, and deteriorate [7,11].

Under the escalating pressures of global climate change and agricultural intensification, these insect pests have evolved remarkable ecological adaptations that enable their survival, reproduction, and expansion [7]. *C*. *formicarius* utilizes a cryptic, endophytic larval habit that provides a spatial refuge, a significant behavioral adaptation, shielding it from extreme environments and conventional surface chemical sprays [8]. In parallel, *B*. *tabaci* exhibits high physiological tolerance to diverse climates, rapid behavioral dispersal, and cross-resistance to major chemical insecticide classes [12]. Currently, management relies heavily on intensive chemical insecticides [6,12]. However, this conventional chemical-centric strategy frequently induces physiological resistance, causes environmental contamination, and fundamentally fails to concurrently manage spatially separated threats [7,11].

Entomopathogenic fungi (EPF), particularly *Beauveria bassiana*, represent a highly promising biological platform for mitigating insect ecological adaptations by acting as robust biopesticides [13,14]. Unlike chemical insecticides, *B*. *bassiana* infects hosts through direct cuticular penetration, effectively circumventing the profound physiological resistance mechanisms that pests have evolved against conventional chemicals. For instance, recent evaluations of wild-type *B*. *bassiana* strains have demonstrated substantial baseline pathogenicity against both highly sclerotized subterranean pests and canopy-dwelling vectors. Specifically, contemporary field and laboratory applications (e.g., strain TMP1) have achieved control efficiencies exceeding 96% against *C*. *formicarius*, significantly reducing storage root yield losses [15,16]. Similarly, recent bioassays have validated the biocontrol efficacy of native *B*. *bassiana* strains (e.g., strain ARP14) against *B*. *tabaci*, demonstrating significant infectivity and mycosis from early instars to adults [17]. However, while demonstrating clear biocontrol potential, wild-type fungal strains often encounter ecological and physiological constraints in complex field applications. They frequently exhibit relatively weak pathogenicity against heavily armored Coleopteran insects and fundamentally lack the trans-order virulence required to maintain high, simultaneous lethality against phylogenetically and ecologically diverse pest complexes. Furthermore, wild-type isolates often suffer from slow infection kinetics or lack the massive sporulation capacity necessary to persist simultaneously in both the exposed canopy microclimate and the subterranean soil profile [16,18].

To overcome these biological bottlenecks, spaceflight mutagenesis has emerged as a cutting-edge technique. Unlike traditional chemical (e.g., ethyl methanesulfonate) or physical (e.g., gamma rays) mutagenesis methods, which often yield lower mutation rates, spaceflight mutagenesis exposes microorganisms to extreme cosmic radiation and microgravity, inducing stable genomic rearrangements and a mutation rate that can be several orders of magnitude higher than terrestrial treatments [19]. This approach induces stable genomic rearrangements, upregulating metabolic pathways associated with enhanced phenotypic plasticity, accelerated virulence, and broad environmental resilience [20]. Accumulating evidence confirms that spaceflight mutagenesis enhances multiple insecticidal traits of EPF, with space-flown isolates inducing significantly higher host mortality than wild-type original strains [21]. Recent studies further validate this paradigm, demonstrating that aerospace environments successfully enhance stress tolerance and phenotypic plasticity in diverse microorganisms, such as increasing the radiation resistance of fungal strains like *Aureobasidium pullulans* [22] and significantly altering the biofilm formation capabilities of bacterial isolates [23]. Spaceflight-mutated elite isolates with enhanced biocontrol traits thus provide core candidate materials for developing this innovative dual-niche pest management paradigm.

By integrating insights from ecology, insect physiology, and molecular biology, this study aims to unravel the potential of spaceflight-induced phenotypic plasticity in EPF to overcome spatially separated pest threats. We report the isolation, molecular identification, and phenotypic characterization of a spaceflight-mutated strain with enhanced virulence, *B*. *bassiana* BbCF-2. We systematically evaluate its dual-niche pathogenicity and lethal kinetics against both the subterranean *C*. *formicarius* and the aboveground vector *B*. *tabaci* to assess its potential for biocontrol. Ultimately, this research evaluates the pathogenicity of a spaceflight-mutated isolate against two representative sweet potato pests.

## 2. Materials and Methods

### 2.1. Parental Strain

The wild-type original strain, designated as *Beauveria bassiana* BbCF-0, was originally isolated from soil collected at Dinghushan, Zhaoqing City, Guangdong, China. This isolate was preserved at the Engineering Research Center of Active Substance Biotechnology, Ministry of Education, Chongqing Normal University, Chongqing, China. For routine culture, the strain was maintained on Potato Dextrose Agar (PDA) (Sangon Biotech (Shanghai) Co., Ltd., Shanghai, China) medium at 26 ± 1 °C in the dark. Conidial suspensions were prepared by harvesting 7-day-old cultures with sterile 0.05% Tween-80 solution (Sangon Biotech (Shanghai) Co., Ltd., Shanghai, China), filtered through two layers of sterile gauze to remove mycelial debris, and adjusted to the required concentration using a hemocytometer.

### 2.2. Spaceflight Mutagenesis Treatment

Spaceflight mutagenesis was performed as described previously with minor modifications. Briefly, 1.0 mL aliquots of standardized *B*. *bassiana* BbCF-0 conidial suspension (1 × 10^7^ conidia/mL) were transferred to sterile polypropylene (PE) centrifuge tubes (Corning Inc., Corning, NY, USA), which were then sealed with Parafilm M (Bemis Company, Inc., Neenah, WI, USA) and placed in specialized spaceflight sample boxes. The samples were launched aboard the ChangZheng 5 space shuttle from the Wenchang Spacecraft Launch Site on 5 May 2020 and remained in low Earth orbit (altitude 300–8000 km) for 67 h. During the flight, the samples were exposed to the combined effects of cosmic radiation, microgravity, and high vacuum and passed through the Van Allen radiation belt multiple times. Due to mission payload constraints, precise environmental parameters such as radiation dose or microgravity exposure were not quantified; this limitation is acknowledged [21].

### 2.3. Mutant Strain Screening and Preservation

After the space capsule returned to Earth, the samples were retrieved and immediately transported to the laboratory under cold chain conditions. The conidial suspension was serially diluted (10^−3^ to 10^−6^) and spread onto PDA plates (100 μL per plate), followed by incubation at 26 ± 1 °C for 7 days. Initially, over 250 mutant colonies were screened. Single colonies exhibiting distinct advantageous traits (rapid radial growth, dense sporulation, and uniform colony morphology) were selected and subjected to 5 consecutive rounds of single-spore isolation to ensure genetic stability.

The elite mutant strain with the most superior biocontrol characteristics (highest sporulation capacity and virulence against both *C*. *formicarius* and *B*. *tabaci*) was formally designated as *B*. *bassiana* BbCF-2. This mutant strain was deposited in the Guangdong Microbial Culture Collection Center (GDMCC) under the accession number GDMCC No. 67196.

### 2.4. Molecular Identification

Genomic DNA was extracted from fresh mycelia of BbCF-2 using the standard CTAB (Sangon Biotech (Shanghai) Co., Ltd., Shanghai, China) method. The internal transcribed spacer (ITS) region of ribosomal DNA was amplified by PCR using the universal primers ITS1 (5′-TCCGTAGGTGAACCTGCGG-3′) and ITS4 (5′-TCCTCCGCTTATTGATATGC-3′). The amplified products were sequenced by Sangon Biotech (Shanghai, China), and the resulting sequence of BbCF-2 was deposited in GenBank under the accession number PV544367. Taxonomic identity was confirmed via BLAST sequence alignment (NCBI BLAST+ v2.14.0, National Center for Biotechnology Information, Bethesda, MD, USA) and Bayesian phylogenetic tree reconstruction (MrBayes v3.2.7, Uppsala University, Uppsala, Sweden) using reference sequences from the NCBI database (Table A1).

### 2.5. Assessment of Biological Productivity and Morphological Traits

To measure the growth rate and conidia yield of BbCF-2, the fungus was cultured on SDAY/4 medium (SDAY/4: 10 g/L dextrose, 2.5 g/L peptone, 2.5 g/L yeast extract, and 15 g/L agar) (Sangon Biotech (Shanghai) Co., Ltd., Shanghai, China) in Petri dishes placed within a biochemical incubator (26 ± 1 °C, 60% RH) for 10 days, and then the conidia were scraped from the plates and suspended in 10 mL of sterile water. Following this, the suspension was filtered through Miracloth (Merck KGaA, Darmstadt, Germany) held in a funnel and quantified using a hemocytometer. The growth rates of BbCF-2 hyphae were measured based on their morphology on an SDAY/4 medium plate on day 10 of culturing. Both examinations were repeated three times.

For the morphometric evaluation of the BbCF-2 isolate, its microcultures were first grown on SDAY/4 and incubated at 27 °C for 10 days. Slides were then prepared with lactophenol/blue cotton (10:1) (Sangon Biotech (Shanghai) Co., Ltd., Shanghai, China) and examined with phase contrast optics under an Olympus BX51 optical microscope (Microscopy GmbH, Gottingen, Germany). Images of the conidia were photographed digitally with an Axio Cam HRc camera (Carl Zeiss AG, Oberkochen, Germany) using the Axion Vision SE64 Release 4.9.1 software.

### 2.6. Insect Rearing and Dual-Niche Target Preparation

Populations representing the integrated aboveground–belowground pest complex were maintained under strictly controlled conditions. A vigorous colony of the subterranean/stem-boring target, *C*. *formicarius*, was continuously reared on sweet potato storage roots within an artificial climate incubator located at the Engineering Research Center. Concurrently, the aboveground foliar target, *B*. *tabaci*, was maintained on host cotton plants within a specialized net room facility. Only active adult stages from both respective pest populations were selected for the subsequent pathogenesis bioassays. Standardized conidial suspensions for these bioassays were prepared by harvesting mature conidia from 7-day-old SDAY/4 cultures into a 0.05% Tween-80 sterile solution. A serial dilution protocol was employed to achieve the precise target concentrations required for toxicological testing.

### 2.7. Pathogenicity Bioassays Against the Subterranean Pest C. formicarius

The lethal kinetics of the BbCF-2 isolate against the root and stem-boring weevil were assessed using a standardized immersion inoculation protocol. Newly emerged *C*. *formicarius* adults were inoculated with six descending concentrations of the BbCF-2 and BbCF-0 conidial suspension: 1 × 10^8^, 1 × 10^7^, 1 × 10^6^, 1 × 10^5^, 1 × 10^4^, and 1 × 10^3^ conidia/mL. The wild-type original strain BbCF-0 was set as the control. A sterile aqueous solution of 0.05% Tween-80 served as the baseline blank control. The experimental matrix consisted of three independent replicates per treatment, with each replicate comprising a cohort of 30 adult weevils. Post-inoculation, the treated cohorts were transferred to a constant-temperature climate chamber. Mortality progression was systematically recorded at 48 h intervals over a comprehensive 15-day observation window. These data were utilized to calculate the corrected cumulative mortality, median lethal concentration (LC50), and median lethal time (LT50).

### 2.8. Pathogenicity Bioassays Against the Aboveground Foliar Pest B. tabaci

To determine the isolate’s efficacy as a canopy intervention, adult *B*. *tabaci* were subjected to foliar spray inoculation utilizing predetermined concentrations of the BbCF-2 and BbCF-0 conidial suspension. The wild-type original strain BbCF-0 was set as the control. Following exposure, the treated whiteflies were securely housed within specialized pathogenicity testing devices designed to simulate the foliar microclimate. Survival trajectories were closely observed and documented every two days post-inoculation. The experimental matrix consisted of three independent replicates per treatment, with each replicate comprising a cohort of 30 adult whiteflies. The resulting temporal mortality dataset was employed to calculate the explicit LC50 value against this sap-sucking vector.

### 2.9. Statistical Analysis

All quantitative datasets derived from the bioassays were subjected to one-way analysis of variance (ANOVA). Prior to ANOVA, proportional mortality data were subjected to an arcsine square root transformation. Assumptions of normality and homogeneity of variance were verified using the Shapiro–Wilk and Levene’s tests (*p* > 0.05). The separation of statistically significant means was executed utilizing Tukey’s multiple-range test via SAS v8.1 software (SAS Institute Inc., Cary, NC, USA). Differences between the two specific strains (BbCF-2 and BbCF-0) in colony growth and sporulation were analyzed using an independent-samples *t*-test. To define the toxicological profile of the BbCF-2 and BbCF-0 isolates, robust probit regression analysis was utilized to calculate both the LC50 and LT50 parameters, inclusive of their respective 95% confidence limits. Mortality data were corrected using Abbott’s formula. The LC50 and LT50 values were estimated utilizing probit regression analysis in SPSS software, assuming a log-normal tolerance distribution (SPSS Statistics v.26 (IBM Corp., Armonk, NY, USA)). All graphical representations modeling survival dynamics and mortality pathways were generated using GraphPad Prism 5 software (GraphPad Software Inc., San Diego, CA, USA).

## 3. Results

### 3.1. Phenotypic Plasticity and Enhanced Biological Productivity Driven by Spaceflight Mutagenesis

**Figure 1:**
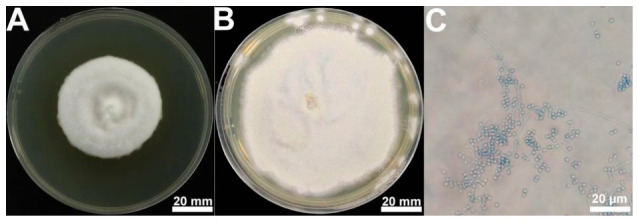
Morphological characterization and aggressive sporulation phenotype of the spaceflight-mutated *Beauveria bassiana* isolate BbCF-2: (**A**) Rapid radial expansion and distinct white, fluffy vegetative colony morphology on SDAY/4 medium at 7 days post-inoculation. (**B**) Mature colony at 14 days, demonstrating a dense, pale-yellow layer indicative of massive sporulation capacity. (**C**) Extensive chains of hyaline, subglobose conidia representing the hypervirulent reproductive structures; microscopic detail of phialides actively generating conidia in characteristic clustered formations.

The mutant isolate BbCF-2, generated via spaceflight mutagenesis, exhibited vigorous vegetative growth, traits essential for counteracting the resilient ecological adaptations of field pests. When cultured on SDAY/4 medium at 26 ± 1 °C, the colony displayed rapid radial expansion, achieving a diameter of 36.2 ± 2.41 mm after 10 days (Table 1). The colony morphology progressed from a dense, fluffy vegetative state to a pale-yellow profile characterized by a uniform sporulation layer (Figure 1A,B). Microscopic evaluation confirmed extensive networks of conidiophores branching in characteristic zigzag patterns, terminating in dense clusters of hyaline, subglobose conidia (Figure 1C).

**Figure 2:**
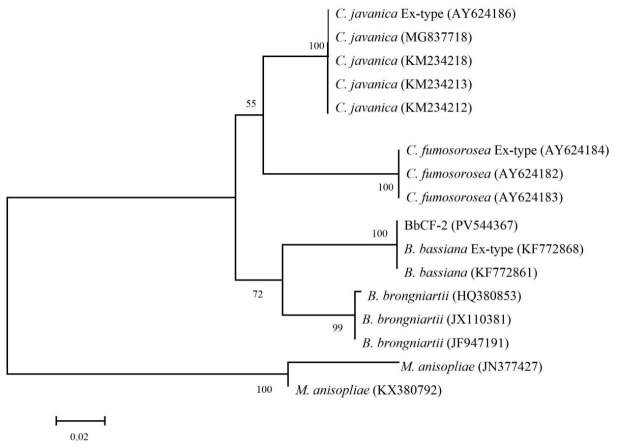
Phylogenetic analysis confirming the taxonomic identity and evolutionary divergence of the mutated isolate BbCF-2. The majority-rule consensus phylogram was constructed using Bayesian inference based on internal transcribed spacer (ITS) sequences. Support values are shown for NJ BS. The distinct clustering of BbCF-2 (GenBank Accession No. PV544367) within the *Beauveria bassiana* clade validates its specific identity while highlighting the genetic stability maintained following spaceflight mutagenesis.

Crucially for commercial biopesticide formulation and large-scale ecological application, the spaceflight-mutated BbCF-2 isolate exhibited both vigorous vegetative growth and an enhanced sporulation capacity. When cultured on SDAY/4 medium at 26 ± 1 °C, the mutant colony displayed significantly faster radial expansion, achieving a diameter of 36.2 ± 2.41 mm after 10 days, compared to only 21.3 ± 1.55 mm for the wild-type progenitor BbCF-0 (69.9% increase; *p* < 0.01). Concurrently, under these standard laboratory conditions, the mutant strain yielded 2.72 × 10^8^ conidia/mL, representing a 74.3% increase in reproductive output compared to the 1.56 × 10^8^ conidia/mL produced by the original strain (*p* < 0.05, Student’s *t*-test) (Table 1). Molecular identification via ITS region sequencing (GenBank Accession No. PV544367) and subsequent Bayesian phylogenetic reconstruction confirms the BbCF-2 mutant within the *B. bassiana* evolutionary clade (Figure 2). This confirms its specific taxonomic identity while highlighting the genetic stability of the advantageous traits acquired through spaceflight mutagenesis.

### 3.2. Overcoming the Physiological Defenses of the Subterranean Pest C. formicarius

**Figure 3:**
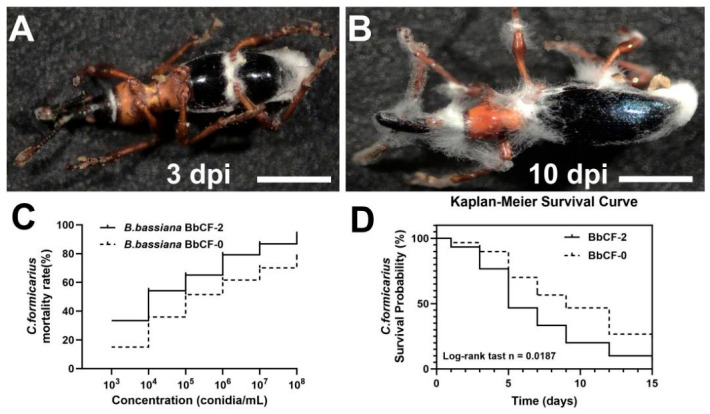
Pathogenicity and morphological infection characteristics of *B. bassiana* BbCF-2 against the subterranean pest, *Cylas formicarius*: (**A**,**B**) Visible mycosis phenotypes of adult weevils treated with a 1 × 10^7^ conidia/mL suspension; the white scale bar represents 2 mm. (**A**) Early-stage infection at 3 days post-inoculation (dpi), highlighting the initial mycelial breach of the highly sclerotized host cuticle. (**B**) Terminal infection stage at 10 dpi, demonstrating massive mycelial outgrowth blanketing the thorax, abdomen, and elytra, confirming the isolate’s capacity for total lethal mycosis. (**C**) Concentration-dependent survival dynamics of *C. formicarius* adults following BbCF-2 exposure and the wild-type BbCF-0 strain. (**D**) Kaplan–Meier survival curves of *C. formicarius* adults treated with BbCF-2 and BbCF-0 (1 × 10^7^ conidia/mL). Each replicate contained 30 adult weevils (*n* = 30 per replicate), with a total of three independent replicates. The log-rank test showed significant differences between survival curves for different strains (*p* < 0.05).

To evaluate its efficacy against the belowground ecological niche, we assessed the virulence of BbCF-2 against adult *C. formicarius*, a species possessing a highly sclerotized cuticle that serves as a formidable physiological defense. BbCF-2 demonstrated highly aggressive host colonization, successfully breaching this physical barrier. Within 48 h post-inoculation, infective hyphae erupted primarily from the cephalic and thoracic intersegmental regions. By day 10, systemic lethal mycosis was evident, with dense mycelial mats entirely engulfing the host’s thorax, abdomen, and elytral veins (Figure 3A,B), visually demonstrating the breakdown of the pest’s structural immunity.

The mutant strain exerted acute, dose- and time-dependent mortality on the weevil populations (Figure 3C). At the maximum deployment concentration of 1 × 10^8^ conidia/mL, cumulative corrected mortality progressed rapidly from 48.67 ± 1.45% on day 5 to an overwhelming 92.68 ± 0.96% by day 15 (Table 2 and Table 3). Toxicological modeling further highlighted the strain’s exceptional capacity to overcome coleopteran resistance, determining a median lethal concentration (LC50) of 8.45 × 103 conidia/mL (95% CI: 3.53 × 10^3^ − 2.02 × 10^4^; r = 0.993) at 15 days post-inoculation (Table 4). Furthermore, Kaplan–Meier survival analysis was employed to quantify the temporal mortality risk. The survival probability of *C. formicarius* adults treated with BbCF-2 declined significantly faster compared to those exposed to the wild-type strain, effectively neutralizing the weevils prior to their critical subterranean oviposition phase (log-rank test, *p* = 0.0187, *n* = 30) (Figure 3D).

### 3.3. Rapid Virulence Against the Canopy-Dwelling Vector B. tabaci

**Figure 4:**
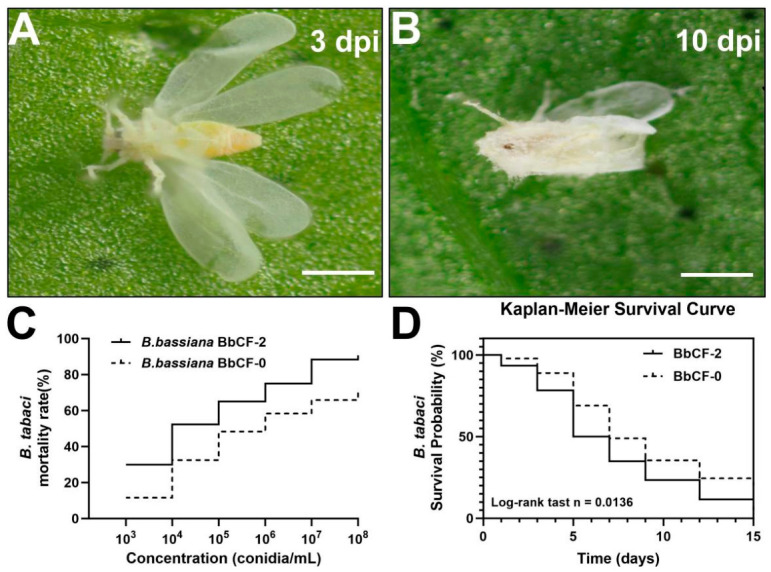
Pathogenicity and morphological infection characteristics of BbCF-2 against the aboveground foliar pest *Bemisia tabaci*: (**A**,**B**) Visible mycosis phenotypes of adult whiteflies treated with a 1 × 10^7^ conidia/mL suspension; the white scale bar represents 500 μm. (**A**) Early-stage infection at 3 dpi, featuring substantial mycelial eruption explicitly from the intersegmental regions. (**B**) Severe terminal infection at 10 dpi, where dense fungal hyphae have completely engulfed the cadaver, a critical trait for facilitating secondary epizootic transmission in the field. (**C**) Concentration-dependent survival dynamics of *B. tabaci* adults following BbCF-2 exposure and the wild-type BbCF-0 strain. (**D**) Kaplan–Meier survival curves of *B. tabaci* adults treated with BbCF-2 and BbCF-0 (1 × 10^7^ conidia/mL). Each replicate contained 30 adult *B. tabaci* (*n* = 30 per replicate), with a total of three independent replicates. The log-rank test showed significant differences between survival curves for different strains (*p* < 0.05).

Operating as a dual-niche biological agent, BbCF-2 simultaneously demonstrated enhanced virulence against the canopy-dwelling vector, *B. tabaci*. The behavioral adaptation of whiteflies, rapid migration and immediate viral transmission, requires a biocontrol agent with acute lethal kinetics. Following foliar exposure, the mutant strain rapidly crashed adult whitefly populations. Postmortem phenotypic analysis confirmed extensive fungal pathogenesis, with BbCF-2 hyphae rapidly emerging from intersegmental regions by 3 dpi to completely consume the cadavers by 10 dpi (Figure 4A,B).

The concentration-dependent survival trajectory collapsed dramatically at high concentrations (Figure 4C). Probit regression analysis underscored the superior biocontrol profile acquired through spaceflight mutation, calculating an acutely low LT50 of 6.718 days for the BbCF-2 isolate against this hemipteran vector. To rigorously evaluate the rapid lethal kinetics required to intercept canopy viral transmission, Kaplan–Meier survival analysis was conducted. The resulting survival curves demonstrated a precipitous and statistically significant reduction in *B. tabaci* survival probability following BbCF-2 exposure compared to the wild-type baseline (log-rank test, *p* = 0.0136, *n* = 30) (Figure 4D). This aggressive infection dynamic supports the BbCF-2 mutant as an elite intervention tool capable of preempting the vector’s dispersal and potential spread of plant viruses.

### 3.4. Temporal Dynamics and Feasibility of the Dual-Niche Strategy

Crucially, a comparative synthesis of the lethal kinetics across both target insect orders establishes the operational feasibility of our integrated management framework to counteract complex ecological adaptations. Under a synchronized application of 1.0 × 10^7^ conidia/mL, the mutant strain delivered an acute median LT50 of 6.718 days (95% CI: 5.396–8.039) against the mobile aboveground vector *B. tabaci* (Table 5). Remarkably, it delivered an even swifter LT50 of 6.305 days (95% CI: 5.014–7.597) against the heavily armored subterranean borer *C. formicarius* (Table 6).

**Figure 5:**
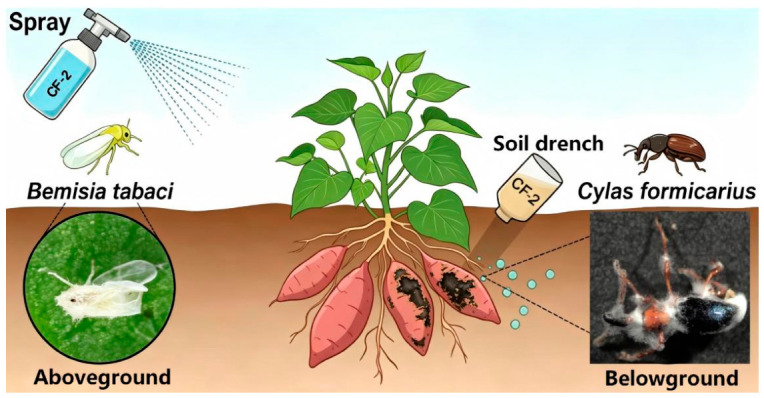
Schematic illustration summarizing the proposed biocontrol strategy of spaceflight-mutated *Beauveria bassiana* BbCF-2 against the sweet potato pest complex. This schematic illustrates a stereoscopic integrated pest management paradigm. Applied foliarly, the hypervirulent BbCF-2 strain induces systemic mycosis in the aboveground viral vector *Bemisia tabaci*; via targeted belowground application, it rapidly kills the root-boring weevil *Cylas formicarius*. This dual-niche intervention eliminates subterranean pest sources, delivers holistic protection to sweet potato storage roots, and provides an innovative, sustainable approach to overcoming the adaptive mechanisms of spatially isolated agricultural pests.

This synchronized temporal progression (Figure 5) demonstrates that a single biological agent can rapidly neutralize canopy virus vectors to suppress foliar damage and, within the exact same chronological window, eliminate robust root-boring adults. These paired toxicological profiles provide the definitive empirical foundation for deploying this spaceflight-mutated strain as a potential candidate against multi-niche pest complexes.

## 4. Discussion

The transition from chemical-reliant agriculture to sustainable integrated pest management necessitates biological control agents capable of resolving complex, multi-niche field challenges. Under the selective pressures of agricultural intensification, the sweet potato pest complex has developed divergent ecological adaptations, notably utilizing spatially separated niches to circumvent conventional monotypic chemical interventions [7,11]. The subterranean root-boring habit of *C*. *formicarius* confers a robust spatial and cuticular barrier [11], whereas the canopy-dwelling *B*. *tabaci* utilizes rapid dispersal mechanisms to facilitate viral transmission [9]. The core achievement of this study is the isolation and validation of the spaceflight-mutated *Beauveria bassiana* strain BbCF-2, which serves as a pleiotropic, multi-niche entomopathogen. Spaceflight-induced microgravity and cosmic radiation are known to trigger genomic rearrangements that significantly upregulate the production of pathogenesis-related secondary metabolites and enzymes, thereby broadening the host range and enhancing the environmental adaptability of entomopathogenic fungi [21]. By demonstrating pronounced trans-order pathogenicity against both Hemipteran and Coleopteran targets, this isolate effectively addresses the spatial divergence and physiological barriers of these pests. Consequently, BbCF-2 shows potential as a foundational biological agent for integrated pest management in sweet potato cultivation, pending comprehensive greenhouse and field evaluations [7,11].

For a biopesticide to be commercially viable across distinct ecological zones (e.g., foliar spraying and soil drenching), it must possess exceptional biological productivity. Recent analyses of commercial mycoinsecticide formulations underscore that baseline sporulation must exceed 10^7^ conidia/mL for economic mass-production feasibility [23,24,25]. Our morphological assays revealed that BbCF-2 exhibits enhanced colony growth, characterized by rapid radial expansion and a high sporulation capacity (2.72 × 10^8^ conidia/mL). This yield is substantially higher than that of wild-type isolates recently evaluated for agricultural deployment, which typically plateau between 3.5 × 10^7^ and 5.0 × 10^7^ conidia/mL under identical solid-state conditions [26]. This superior reproductive trait is a hallmark of successful spaceflight mutagenesis. Microgravity and cosmic radiation have been shown to induce stable genomic rearrangements that upregulate metabolic pathways related to biomass accumulation, such as the amplification of chitin synthase expression, directly correlating with enhanced environmental resilience and conidial yield [19]. Such engineered biological productivity is the primary prerequisite for the cost-effective formulation required to execute large-scale, dual-niche applications.

Against the subterranean threat of *C*. *formicarius*, rapid cuticular degradation is imperative for efficient pest mortality [11,18,27]. The BbCF-2 isolate exhibited an exceptionally rapid median lethal time (LT50) of 6.305 days, significantly outperforming the sluggish kinetics (LT50: 8.4–11.2 days) typical of indigenous soil isolates against coleopteran pests, although some specific hypervirulent *Metarhizium anisopliae* isolates have achieved high mortality within 7 days [28]. The aggressive emergence of mycelia from highly sclerotized thoracic joints phenotypically demonstrates an enhanced capacity for physical host colonization [29]. This enhanced virulence phenotype is consistent with the toxicological characteristics of previously reported spaceflight-mutated entomopathogenic fungi; a space-bred *Purpureocillium lilacinum* strain reduced its LT50 against *Tetranychus cinnabarinus* by 78.8% relative to the wild type, while BbCF-2 achieved a 37.7% LT50 reduction against *C*. *formicarius*, reflecting species-specific responses to aerospace mutagenesis [21].

Equally compelling is BbCF-2′s acute pathogenesis against the aboveground canopy vector, *B*. *tabaci*. Whiteflies exploit the behavioral adaptation of rapid population surges and immediate migration to execute viral transmission; thus, mitigating viral spread strictly requires highly virulent strains capable of inducing rapid lethal mycosis before the insects disperse [30,31,32]. The median lethal time (LT50) of BbCF-2 against this hemipteran vector was acutely accelerated, achieving 50% mortality in just 6.718 days at an inoculation rate of 1.0 × 10^7^ conidia/mL, which represents a significantly faster lethal kinetic compared to the 10.215 days required by our wild-type reference strain. Furthermore, this rapid pathogenesis compares highly favorably against widely commercialized *B*. *bassiana* strains (e.g., strain GHA), which typically exhibit LT50 values exceeding 8 to 10 days against Bemisia populations under comparable application rates [33]. This acute hypervirulence ensures that direct foliar applications can precipitate rapid population collapse, thereby disrupting the viral transmission cascade.

Managing the sweet potato pest complex requires moving beyond isolated, single-pest interventions. The spaceflight-mutated BbCF-2 effectively bridges the gap between canopy and soil pest management. Its unique combination of phenotypic plasticity (commercial-scale sporulation), potent physiological mechanisms (rapid cuticular penetration against Coleopteran defenses), and acute hypervirulence against Hemipteran vectors makes it a premier candidate for integration into the sustainable green management system (SGMS) framework [7]. Future field efficacy trials evaluating dual-delivery mechanisms, such as simultaneous foliar application targeting vector populations and micro-drip soil irrigation targeting subterranean physical defenses, will further unlock the aboveground–belowground protective potential of this elite engineered isolate.

### Future Perspectives

While the empirical data demonstrate the biocontrol potential of BbCF-2, the underlying mechanisms require further investigation. Emerging paradigms suggest spaceflight-induced virulence may rely on the time-dependent downregulation of detoxifying and antioxidant enzymes, alongside microbiota destabilization [21]. Validating this enzymatic and transcriptomic interplay remains a primary objective. Additionally, translating this potential into field efficacy may involve integrating BbCF-2 with specific sex pheromones in an ‘attract-and-infect’ paradigm [34,35,36]. Furthermore, deploying BbCF-2 as a soil drench warrants investigation into potential secondary mechanisms, such as plant-mediated systemic resistance and endophytic mutualism [37,38,39].

## Figures and Tables

**Table 1:** Colony expansion dynamics and mass-sporulation capacity of the spaceflight-mutated *Beauveria bassiana* BbCF-2 and BbCF-0 isolates on SDAY/4 medium (10 days post-inoculation).

Strain	Colony Diameter Expansion ± SE (mm)	Sporulation (Conidia/mL)
BbCF-2	36.2 ± 2.41 **	2.72 × 10^8^ *
BbCF-0	21.3 ± 1.55	1.56 × 10^8^

Note: Data are presented as mean ± standard error (SE) of three independent replicates. Asterisks indicate highly significant differences between the mutated isolate BbCF-2 and the wild-type original strain BbCF-0 according to Student’s *t*-test (* *p* < 0.05; ** *p* < 0.01).

**Table 2:** Concentration-dependent pathogenic efficacy of *B. bassiana* BbCF-2 and BbCF-0 against newly emerged adult sweet potato weevils (*Cylas formicarius*) at 15 days post-inoculation.

Concentration (Conidia/mL) ± SE (%)	Corrected Cumulative Mortality of *C. formicarius* Adults M ± SE (%)	
	BbCF-2	BbCF-0
1 × 10^8^	92.68 ± 0.96 a	78.12 ± 0.61 a
1 × 10^7^	90.07 ± 0.44 a	70.14 ± 1.34 b
1 × 10^6^	79.33 ± 1.20 b	61.23 ± 1.68 c
1 × 10^5^	65.11 ± 2.15 c	52.00 ± 1.20 d
1 × 10^4^	54.00 ± 1.53 d	36.00 ± 2.00 e
1 × 10^3^	33.33 ± 1.45 e	14.67 ± 0.88 f

Note: Data are presented as mean ± SE. Same letters in columns mean insignificant differences in these variants. Tukey test *p* < 0.05.

**Table 3:** Temporal dynamics of cumulative corrected mortality in *C. formicarius* adults subjected to *B. bassiana* BbCF-2 and BbCF-0 infection.

Time (d) ± SE (%)	Cumulative Corrected Mortality of *C. formicarius* Adults M ± SE (%)	
	BbCF-2	BbCF-0
1	8.00 ± 1.15 f	1.00 ± 0.58 g
3	23.00 ± 1.63 e	10.04 ± 1.15 f
5	48.67 ± 1.45 d	28.33 ± 0.67 e
7	63.33 ± 0.88 c	42.67 ± 1.76 d
9	76.67 ± 2.03 b	54.00 ± 2.01 c
12	89.63 ± 0.71 a	71.67 ± 0.88 b
15	92.68 ± 0.96 a	78.12 ± 0.61 a

Note: Data are presented as mean ± SE. Same letters in columns mean insignificant differences in these variants. Tukey test *p* < 0.05.

**Table 4:** Toxicological parameters (LC50) defining the lethal concentration threshold of *B. bassiana* BbCF-2 against the subterranean/stem-boring pest *C. formicarius*.

Parameter	BbCF-2
Day	15
Regression Equation	Y = 0.383X + 3.496
LC_50_ (Conidia/mL)	8.452 × 10^3^
95% Confidence Interval (Conidia/mL)	(3.532 × 10^3^ − 2.0224 × 10^4^)
Correlation Coefficient (r)	0.993

**Table 5:** Comparative virulence assessment (LT50) confirming the enhanced lethality of spaceflight-mutated *B. bassiana* BbCF-2 versus the reference strain BbCF-0 against the foliar vector *B. tabaci*.

Parameter	BbCF-2	BbCF-0
Inoculum Concentration	1.0 × 10^7^	1.0 × 10^7^
Regression Equation	Y = 0.196X + 3.678	Y = 0.205X + 2.901
LT_50_ (Days)	6.718	10.215
95% Confidence Interval (Days)	(5.396–8.039)	(8.231–12.198)
Correlation Coefficient (r)	0.971	0.952

**Table 6:** Lethal kinetics (LT50) demonstrating the rapid infection timeline of *B. bassiana* BbCF-2 and BbCF-0 against adult *C. formicarius*.

Parameter	BbCF-2	BbCF-0
Inoculum Concentration	1.0 × 10^7^	1.0 × 10^7^
Regression Equation	Y = 0.204X + 3.713	Y = 0.1963X + 3.165
LT_50_ (Days)	6.305	9.351
95% Confidence Interval (Days)	(5.014–7.597)	(7.705–10.997)
Correlation Coefficient (r)	0.973	0.961

## Data Availability

The original data are included in the paper. Further inquiries can be directed to the corresponding authors.

## Abbreviations

The following abbreviations are used in this manuscript:

*B. bassiana*	*Beauveria bassiana*
*C. formicarius*	*Cylas formicarius*
*B. tabaci*	*Bemisia tabaci*
LC_50_	Lethal concentration 50
LT_50_	Lethal time 50
BbCF-0	The wild-type original strain of *B. bassiana*
BbCF-2	The elite mutant strain of *B. bassiana*

## Appendix A

**Table A1:** The reference entomopathogenic fungi from GenBank used in phylogenetic analysis. (“*” is the GenBank accession number of BbCF-2; “N/A”, unlabeled in NCBI).

Species	GenBank Number	Strain No.	Host	Location
*Cordyceps javanica*	AY624186	Ex-type CBS 134.22	*Hypothenemus hampei*	Java
*Cordyceps javanica*	MG837718	ACP	*Diaphorina citri*	Fuzhou, China
*Cordyceps javanica*	KM234218	CHE-CNRCB 307/7	*Bemisia tabaci*	Armeria, Mexico
*Cordyceps javanica*	KM234213	CHE-CNRCB 303/2	*Bemisia tabaci*	Armeria, Mexico
*Cordyceps javanica*	KM234212	CHE-CNRCB 303	*Bemisia tabaci*	Armeria, Mexico
*Cordyceps* *fumosorosea*	AY624182	CBS 244.31	Butter	Ireland
*Cordyceps* *fumosorosea*	AY624183	CBS 375.70	Food	Japan
*Cordyceps* *fumosorosea*	AY624184	Ex-type CBS 107.10	*N/A*	France
*Beauveria bassiana*	PV544367	BbCF-2 *	soil	Guangdong, China
*Beauveria bassiana*	KF772868	GA-1	*Micromelalopha troglodyta*	Hubei, China
*Beauveria bassiana*	KF772861	YD-1	*Dendroctonus punctatus*	Hubei, China
*Beauveria brongniartii*	HQ380853	ART376	*Melolontha melolontha*	Innerkirchen, Switzerland
*Beauveria brongniartii*	JX110381	SASR HHB32B	*Galleria mellonella*	South Africa
*Beauveria brongniartii*	JF947191	ARSEF8153	*Agrilus planipennis*	Ontario, Canada
*Metarhizium anisopliae*	JN377427	ZJ	*Rhynchophorus ferrugineus*	Hainan, China
*Metarhizium anisopliae*	KX380792	YD2-1-8	soil	Nanchang, China
